# Facts and myths about the use of lithium for bipolar disorder in routine clinical practice: an expert consensus paper

**DOI:** 10.1186/s12991-023-00481-y

**Published:** 2023-12-06

**Authors:** Andrea Fiorillo, Gaia Sampogna, Umberto Albert, Giuseppe Maina, Giulio Perugi, Maurizio Pompili, Gianluca Rosso, Gabriele Sani, Alfonso Tortorella

**Affiliations:** 1 Department of Psychiatry, University of Campania “L. Vanvitelli”, Largo Madonna delle Grazie, Naples, Italy; 2 Department of Medicine, Surgery and Health Sciences, University of Trieste, Trieste, Italy; 3 San Luigi Gonzaga Hospital, University of Turin, Turin, Italy; 4 Department of Neurosciences “Rita Levi Montalcini”, University of Turin, Turin, Italy; 5 Department of Clinical and Experimental Medicine, University of Pisa, Pisa, Italy; 6 Department of Neurosciences, Mental Health and Sensory Organs, Suicide Prevention Center, Sant’Andrea Hospital - Sapienza University of Rome, Rome, Italy; 7 Department of Neuroscience, Section of Psychiatry, Università Cattolica del Sacro Cuore, Largo Francesco Vito 1, 00168 Rome, Italy; 8 Department of Psychiatry, Fondazione Policlinico Universitario Agostino Gemelli IRCCS, Largo Agostino Gemelli 8, 00168 Rome, Italy; 9 Department of Psychiatry, University of Perugia, Perugia, Italy

**Keywords:** Lithium, Bipolar disorder, Pharmacological treatment, Side effect, Neuroprotection

## Abstract

**Background:**

Bipolar disorder is one of the most burdensome severe mental disorders, characterized by high levels of personal and social disability. Patients often need an integrated pharmacological and non-pharmacological approach. Lithium is one of the most effective treatments available not only in psychiatry, but in the whole medicine, and its clinical efficacy is superior to that of other mood stabilizers. However, a declining trend on lithium prescriptions has been observed worldwide in the last 20 years, supporting the notion that lithium is a ‘forgotten drug’ and highlighting that the majority of patients with bipolar disorder are missing out the best available pharmacological option.

Based on such premises, a narrative review has been carried out on the most common “misconceptions” and “stereotypes” associated with lithium treatment; we also provide a list of “good reasons” for using lithium in ordinary clinical practice to overcome those false myths.

**Main text:**

A narrative search of the available literature has been performed entering the following keywords: “bipolar disorder”, “lithium”, “myth”, “mythology”, “pharmacological treatment”, and “misunderstanding”. The most common false myths have been critically revised and the following statements have been proposed: (1) Lithium should represent the first choice for the treatment of patients with bipolar disorder; (2) lithium treatment is effective in different patients’ groups suffering from bipolar disorder; (3) Drug–drug interaction risk can be easily managed during lithium treatment; (4) The optimal management of lithium treatment includes periodical laboratory tests; (5) Slow-release lithium formulation has advantages compared to immediate release formulation; (6) Lithium treatment has antisuicidal properties; (7) Lithium can be carefully managed during pregnancy.

**Conclusions:**

In recent years, a discrepancy between evidence-based recommendations and clinical practice in using lithium treatment for patients with bipolar disorder has been highlighted.

It is time to disseminate clear and unbiased information on the clinical efficacy, effectiveness, tolerability and easiness to use of lithium treatment in patients with bipolar disorder. It is necessary to reinvigorate the clinical and academic discussion about the efficacy of lithium, to counteract the decreasing prescription trend of one of the most effective drugs available in the whole medicine.

## Background

Bipolar disorder is one of the most burdensome severe mental disorders, characterized by high levels of personal and social disability [1]. Patients often need an integrated [2–4] and personalized [5, 6] pharmacological and non-pharmacological approach [7, 8]. In particular, when an acute depressive, manic or mixed episode occurs, a pharmacological treatment is usually needed; however, the majority of bipolar patients need long-term treatments, to prolong the free interval, to prevent recurrences, to lessen subthreshold symptoms, to improve relational, social, and occupational functioning [6, 9]. Treatment may be complicated by the presence of comorbid physical illnesses, such as cardiovascular diseases, metabolic syndromes (i.e., type 2 diabetes and obesity) [10–12], and other mental disorders, such as alcohol or substance disorder, anxiety disorders and personality disorders [13–19].

Several pharmacological treatments are indicated as first-line treatment for the management of patients with bipolar disorder, with lithium being the gold standard due to its established mood-stabilizing properties, its effectiveness in preventing recurrences, and its anti-suicidal effects [20, 21]. Since its introduction more than 70 years ago [22], lithium has been successfully used to treat bipolar disorder, and it is now unanimously considered the first line option for acute and long-term treatment [23, 24]. Lithium is also highly effective in other serious mental disorders characterized by internalizing and externalizing behaviours, due to its anti-impulsivity and anti-suicidality effects.

Lithium is one of the most effective treatments available not only in psychiatry, but in the whole medicine [25], and its clinical efficacy is superior to that of other mood stabilizers. However, a declining trend on lithium prescriptions has been observed worldwide in the last 20 years, supporting the notion that lithium is a ‘forgotten drug’ [26] and highlighting that the majority of BD patients are missing out the best available pharmacological option [27].

This decreasing trend in the use of lithium in clinical practice can be due to several factors, including its narrow therapeutic index, the side-effect/tolerability profile, the need for regular blood checking [28], the preference of many psychiatrists—particularly the less experienced ones—to use other mood stabilizers which are commonly considered more manageable, safer and less toxic [29]. However, this reflects an important incongruity between evidence-based recommendations and psychiatric clinical practice, and is probably due to the presence of several “false myths” about lithium use among physicians [21, 28], patients and their family members. Providing appropriate education might reverse this concerning trend. Thus, efforts in improving training on lithium should represent a priority for postgraduates and residents around the world in the next years [30].

Based on such premises, we carried out a narrative review of the available literature on the most common “misconceptions” and “stereotypes” associated with lithium treatment; for each false myth, we provide a list of “good reasons” for using lithium in ordinary clinical practice.

## Methods

The keywords “bipolar disorder”, “lithium”, “myth”, “mythology”, “pharmacological treatment”, and “misunderstanding” were entered in PubMed, ISI Web of Knowledge, Scopus and Medline. Terms and databases were combined using the Boolean search technique, which consists of a logical information retrieval system (two or more terms combined to make searches more restrictive or detailed).

**Fig. 1:**
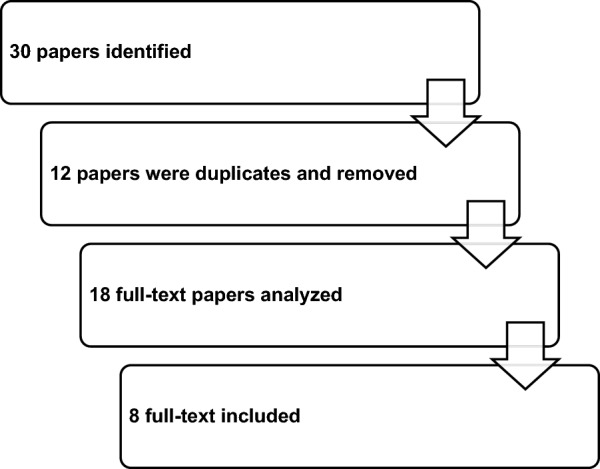
Selection process of included papers in the narrative review

All selected papers were evaluated by two authors (GS and AF); main findings of included papers are summarized in Fig. 1 and in Table 1.

**Table 1:** Narrative review of the literature

Author(s), year, country	Title	Journal	Main statement/main findings
Malhi, 2021, US [31]	Lithium mythology	Bipolar disorder	Lithium is an old drug; it has nothing new to offer Lithium seldom works Lithium is not suitable first line Lithium is complicated to prescribe and manage Lithium is a dirty drug and difficult to tolerate Lithium destroys thyroid function Lithium ruins kidney function and eventuates in renal failure
Zivanovic, 2017, Serbia [156]	Lithium: A classic drug-Frequently discussed, but, sadly, seldom prescribed!	Australian New Zealand Journal Psychiatry	The evidence of the effectiveness of lithium in the treatment of acute mania, acute bipolar depression and the prevention of manic and depressive episodes is compelling. Lithium is the most effective augmentation agent in treatment-resistant depression. Its anti-suicidal effects are well established. The neuroprotective effects of lithium have been demonstrated in case–control studies and in population-based research. It has been established that starting lithium early in the course of the disorder reduces the rates of treatment non-response
Fawcett, 1994, US [32]	Some Provocative Thoughts About Continuation and Maintenance Treatment	Psychiatric Annals	Lithium is effective in treating bipolar disorder in the long-term
Schou, 2003, US [33]	Lithium Prophylaxis: Myths and Realities	Focus	Effect in rapid cyclers Long-term effects on thyroid function Long-term effects on renal function Frequency of side effects Effect on creativity Development of intoxication with Therapeutic doses and serum levels Risk involved in combining lithium and neuroleptics Prevalence of lithium use
Sachs et al., 1994, US [34]	Lithium monotherapy: Miracle, myth and misunderstanding	Psychiatric Annals	The early use of LI as a "miracle" drug is documented. The myth of these miracle properties is dispelled, and LI's strengths and weaknesses are realistically discussed. LI is ineffective for rapidly cycling bipolar patients, and its antidepressant effects are modest
Schou, 1989, US [35]	Lithium prophylaxis: Myths and Realities	American Journal of Psychiatry	Effect in rapid cyclers Long-term effects on thyroid function Long-term effects on renal function Frequency of side effects Effect on creativity Development of intoxication with Therapeutic doses and serum levels Risk involved in combining lithium and Neuroleptics Prevalence of lithium use
Shepherd, 1970, US [36]	A prophylactic myth	Int J Psychiatry	Lithium is effective in preventing relapses in patients with bipolar disorder, but has several side effects
Blackwell & Shepherd, 1968, US [37]	Prophylactic lithium: another therapeutic myth?	Lancet	The concept of "prophylactic" lithium was first formulated from the grouping of individuals maintained on the drug for long periods. They confirmed that lithium is effective in preventing relapses

## Results from the narrative clinical review

Based on the search strategy, 30 papers were identified; 12 papers were removed because duplicates; 18 full-text papers were fully analyzed, and eight papers were finally included in the review (Fig. 1).

The majority of studies were carried out in the US [31–37] in the period 1968–2021 and published in peer-reviewed international scientific journals, such as the American Journal of Psychiatry or Bipolar Disorder (Table 1).

The most common false myths, which have been critically revised, are the following: (1) lithium is not the first choice for treating patients with bipolar disorder; (2) lithium should be avoided in adolescents or elderly patients due to its side effects; (3) the risk of drug–drug interactions is one of the most common limitations in lithium treatment; (4) weekly lab tests are required during treatment with lithium; (5) different lithium formulations do not modify its tolerability profile; (6) no drug has antisuicidal effects; (7) lithium should be avoided during pregnancy (Table 2). The following statements, based upon the most recent evidence on lithium treatment, are proposed: (1) lithium should represent the first choice for the treatment of patients with bipolar disorder; (2) lithium treatment is effective in different patients’ groups, including young and elderly patients; (3) the risk of drug–drug interaction during lithium treatment can be easily managed; (4) the optimal management of lithium treatment includes periodical laboratory tests; (5) slow-release lithium formulation has advantages compared to immediate release formulation; (6) lithium has an antisuicidal effect; (7) lithium can be carefully managed during pregnancy.

**Table 2:** Myths and facts about lithium treatment in psychiatric practice

Myth	Fact
Lithium is not the first choice in bipolar disorder	Lithium should represent the first choice for the treatment of patients with bipolar disorder
Lithium should be avoided in adolescents and elderly patients	Lithium treatment is effective in different patients’ groups with bipolar disorder, including young and elderly patients
The risk of drug–drug interactions is an important limitation in lithium treatment	The risk of drug–drug interaction can be easily managed during lithium treatment
Weekly lab tests are required during treatment with lithium	The optimal management of lithium treatment includes periodical laboratory tests
Different lithium formulations do not modify its tolerability profile	Slow-release lithium formulation has advantages compared to immediate release formulation
No drug has antisuicidal effects	Lithium has an antisuicidal effect
Lithium should be avoided during pregnancy	Lithium can be carefully managed during pregnancy

### Myth 1: lithium is not the first choice for treating patients with bipolar disorder

#### Fact 1: lithium should represent the first choice for the treatment of patients with bipolar disorder

Lithium plays a relevant role in acute and long-term management of bipolar disorder and must considered as first-line treatment [38]. In fact, lithium increases the duration of free intervals, minimizes the risk of recurrences and improves inter-episodic symptomatology. Nowadays, lithium is still the gold standard in studies evaluating the efficacy of various medications in the long-term treatment of recurrent mood disorders [39].

Available evidence strongly indicates that patients suffering from bipolar disorder should be primarily treated with lithium, using other mood stabilizers as add-on in case of partial response [40]. Moreover, lithium treatment should be started as early as possible, since response rates for mania and for long-term treatments decrease in individuals with more than three episodes [41]. Maintenance treatment with lithium should be started after two hypomanic episodes or even after one severe psychotic, manic or mixed episode [42].

Patients should be educated about the many benefits of a long-term treatment with lithium, in terms of prophylaxis of mood episodes, reduction of suicidal risk, and neuroprotective effects, with a probable reduction of the risk of dementia and a potential protection against cognitive impairment that is a long-term consequence of multiple mood episodes [43].

### Myth 2: lithium should be avoided in adolescents or elderly patients due to its side effects

#### Fact 2: lithium treatment is effective in different patients’ groups, including young and elderly patients

Lithium represents the gold standard for the treatment of adult with bipolar disorder, but its role in treating bipolar disorder in childhood or adolescence is still debated.

Amerio et al. [44] concluded that lithium monotherapy is safe and effective for acute mania and for the prevention of affective episodes in children and adolescents. A recent umbrella review highlighted that lithium is reasonably safe and effective in children and adolescents, with adverse events similar to those observed in adults; however, the authors underlined that available evidence is limited, and further studies are needed [45].

Optimal dosing strategies have been extensively studied in the pediatric literature. In children weighing > 30 kg, due to the shorter half-life elimination and the greater creatinine clearance, the dosing strategy with lithium begins at a dose of 300 mg daily, followed by a 300 mg weekly increase, until it reaches serum lithium levels ranging from 0.8 to 1.2 mmol/l, similar to those of adults [46]. This strategy yielded mean total daily doses of 1500 mg (SD = 400.9 mg) and a mean weight-adjusted total daily dose of 29.1 mg/kg/day.

According to the Systematic Treatment Enhancement Program for Bipolar Disorder (STEP-BD) study, lithium is prescribed more frequently to adult (37.8%) than to elderly bipolar patients (29.5%) [47]. However, because elderly subjects are often excluded from randomized clinical trials, studies focused on the treatment of bipolar disorder in older age are lacking and the information is mainly based on data derived from mixed age populations [48, 49]. A growing attention is being given to a subset of patients with bipolar disorder, defined “older age bipolar disorder” (OABD), i.e., bipolar patients aged 50 years and over with prevalent cognitive deficits, increased risk of dementia, impaired psychosocial functioning, frequent physical comorbidities, and premature death [50, 51]. In a double-blind, randomized, controlled trial in elderly patients with bipolar disorder, lithium was more effective than valproate in reducing manic symptoms during a 9 week follow-up and both drugs were similarly well tolerated [52].

Recently, it has been proposed that optimal serum levels of lithium in elderly patients between 60 and 79 years should be 0.4–0.8 mmol/l, while in patients aged 80 or more lithium levels should be between 0.4 and 0.7 mmol/l [53]. In the case of physical comorbidities, polypharmacy, cerebrovascular diseases, parkinsonism and dementia, a serum lithium concentration equal to or lower than 0.5 mmol/l (measured after 12 h) is recommended [54].

A dose reduction of lithium of about 20% is often required in elderly patients compared to younger patients. However, for maintenance monotherapy in OABD lithium is effective and well tolerated and it still represents the preferred choice, if correctly used. However, special caution is required to prevent nephropathy and intoxication.

In the elderly, lithium seems to be used more frequently as add-on to antidepressants in the treatment of resistant depression than in bipolar disorder [55]. Long-term treatment with lithium in the elderly is effective and relatively well tolerated both in patients with bipolar disorder and in those suffering from resistant depression. Morlet et al. [56] found that patients with depressive and bipolar disorder who had taken lithium in previous years (12.5 years on average) had less severe psychiatric symptoms, less severe depressive symptoms, and less use of benzodiazepines compared to those who had not received lithium therapy. Except for hypothyroidism, patients taking lithium have no more side effects compared to those not taking lithium.

### Myth 3: the risk of drug–drug interactions represents one of the most common limitations in lithium treatment

#### Fact 3: drug–drug interaction risk can be easily managed during lithium treatment

Drug–drug interactions with lithium can be pharmacokinetic or pharmacodynamic in nature.

As regards pharmacokinetic interactions, lithium has a narrow therapeutic index and changes in plasma concentrations can have significant clinical consequences. The ion is extensively absorbed in the gastrointestinal tract, is not metabolized and is almost entirely eliminated by the kidneys [57]. Serum lithium levels are sensitive to physiological factors that affect renal function, including age, dehydration, sodium balance; the most important drug interactions occur when co-administered drugs alter renal function, specifically modifying glomerular filtration and tubular reabsorption.

The most commonly prescribed drugs that have the potential to interact with lithium are ACE inhibitors, angiotensin-II receptor antagonists (sartans), diuretics, and non-steroidal anti-inflammatory drugs (NSAIDs) [58] (Table 3).

**Table 3:** Lithium drug–drug interactions

Pharmacokinetics	
**Increase lithium concentrations**	
Angiotensin-converting enzyme (ACE) inhibitors	
Angiotensin-II receptor antagonists	
Diuretics: thiazides, spironolactone, furosemide	
Non-steroidal anti-inflammatory drugs (NSAIDs)	
Other: metronidazole, baclofen, cotrimoxazole, aciclovir, prostaglandin-synthetase inhibitors tetracyclines, topiramate	
**Decrease lithium concentrations**	
Sodium bicarbonate and sodium chloride containing products	
Xanthines: theophylline, caffeine	
Other: urea, mannitol, acetazolamide	
Pharmachodynamic (Neurotoxicity)	
Antipsychotics (FGA and SGA)	Tremor, extrapyramidal symptoms, myoclonus Confusion, disorientation, lethargy, NMS Supersensitivity (Tardive Diskinesia, rebound psychosis)
SSRIs, sumatriptan,	Tremors, dizziness, agitation, confusion, or diarrhea Serotonin syndrome
Calcium channel blockers	Ataxia, confusion and somnolence, reversible after discontinuation of the medicine
Carbamazepine, phenytoin	Dizziness, somnolence, confusion, cerebellar symptoms
Methyldopa	Confusion, disorientation and hand tremors
Neuromuscular blocking agents	Lithium may prolong the effects of these agents
Electro Convulsive Therapy	Delirium

Case reports and hospital admission studies have shown that ACE inhibitors and angiotensin-II receptor antagonists can increase lithium serum concentrations, thus increasing the risk of toxicity [59]. Closer monitoring of lithium concentrations is needed when people start either of these drugs, and lithium dose probably needs to be reduced until a stable therapeutic concentration has been achieved. Closer monitoring for several days is also required when those drugs are stopped.

Lithium concentrations must be carefully monitored when a diuretic drug is prescribed. Thiazide and thiazide-like diuretics increase sodium reabsorption, which decreases the clearance of lithium and significantly elevates its serum concentrations [60]. Amiloride is recommended as a diuretic because of its mechanism of action that reduces lithium accumulation and improves kidney function in long-term treatment [61].

Patients taking lithium should be advised not to use regularly nonsteroidal anti-inflammatory drugs (NSAIDs) that can alter lithium concentrations through multiple mechanisms [62]. If NSAIDs are indicated, they should be used under medical guidance with a close monitoring of lithium concentrations; in these cases, lower lithium doses may be required.

Accelerating lithium elimination can be obtained through decreasing lithium reabsorption in the proximal tubule by osmotic diuresis (e.g., mannitol), carbonic anhydrase inhibitor, acetazolamide, and sodium bicarbonate [63]. Some calcium-channel blockers, such as nifedipine or nimodipine, can increase lithium clearance by producing afferent arteriolar vasodilatation. A similar effect is produced by xanthines, such as aminophylline, theophylline, and caffeine. Abrupt withdrawal from excessive drinking of coffee or tea may decrease lithium clearance, which may result in intoxication [57].

When combining lithium with other mood stabilizers, such as carbamazepine, valproate or lamotrigine, there is no significant influence on the levels of each drug; furthermore, combinations with tricyclic antidepressants are pretty safe [64].

Increased lithium neurotoxicity can be caused by the interaction with many drugs, particularly if they are administered at high doses and in elderly patients. These drugs include first (chlorpromazine and other phenothiazines, haloperidol) and second-generation antipsychotics (clozapine, olanzapine, quetiapine, risperidone, paliperidone, aripiprazole, and brexpiprazole). Lithium and antidopaminergic drugs can induce a profound dopamine hypofunctionality as a causative mechanism for neurotoxicity, resulting in increased risk of extrapyramidal symptoms, neuroleptic malignant syndrome and tardive dyskinesia [65].

Other interactions include the enhancement and prolongation of the action of competitive (e.g., pancuronium) and depolarizing (succinylcholine) muscle relaxants which, in rare cases, can trigger attacks of congenital muscle fatigue [66].

Patients with bipolar disorder often receive specific medications for treating comorbid physical conditions, such as obesity, hypertension and cardiovascular disorders. Therefore, interactions between lithium and those medications are frequent.

In patients with high blood pressure treated with certain diuretics, i.e., thiazide diuretics, ACE-inhibitors, and angiotensin-II receptor antagonists, or undergoing low sodium diet, serum levels of lithium might increase up to toxic concentrations. Therefore, different therapeutic options, i.e., loop diuretics, should be considered in these patients [67].

Although caution is required when prescribing lithium to subjects with cardiovascular diseases, such as arrythmias and QT prolongation, especially with concomitant electrolyte imbalances [53], Ponzer et al. [51] found a lower risk for cardiovascular and cerebrovascular diseases in BD patients receiving lithium.

Since lithium pharmacokinetics and body distribution change with body weight, larger maintenance doses may be required in obese patients. Therefore, a strict monitoring of the onset of potential side effects of lithium [68] is recommended.

According to the most recent recommendations, a target serum lithium concentration range of 0.5–0.8 mmol/L (varying upon clinical indication, age, and concurrent physical status) seems most appropriate for most patients. Lower end levels of the therapeutic range (0.5–0.6 mmol/L) are generally recommended for older patients (50 years and over) and for those taking interacting concomitant medications for other risk factors such as heart disease, renal impairment, diabetes insipidus, thyroid dysfunction [67].

Lithium should be prescribed with caution in patients receiving drugs that can slow its renal elimination and increase the risk of toxic effects (e.g., thiazide diuretics, ACE-inhibitors, angiotensin converting enzyme inhibitors, NSAIDs, when assumed more than occasionally and for extended periods), and if a low-sodium diet is required for medical reasons [53].

Kuramochi et al. [69] investigated the administration rates of NSAIDs, loop/thiazide diuretics, angiotensin-converting enzyme inhibitors, and/or angiotensin-II receptor blockers between lithium users and age- and sex-matched non-lithium users. They also investigated the number of patients in the two groups with a diagnosis of somatic conditions who were receiving those medications. Results show that prescriptions of the above medications are less frequent in lithium users compared to non-users (18.3 vs. 31.9%), with subsequent suboptimal treatment of medical comorbidities and impact on physical health.

The presence of alcohol use disorders (AUD) and substances use disorders (SUD) should always be considered when initiating a treatment with lithium [70, 71]. Lithium is still considered the first-line treatment in comorbid BD-AUD/SUD patients who show good adherence. Also, medications for AUD can be safely used in BD given the lack of significant pharmacological interactions [72]. However, in these patients the combination of more drugs is often the rule for improving patients’ outcome. For example, in patients affected by bipolar disorder with AUD/SUD, adding an anticonvulsant drug to lithium is preferable to lithium monotherapy for their effects on substance consumption and craving [73]; the combination of lithium and valproate is effective for affective symptoms and reduces substance use, possibly through an indirect effect on mood stability [74]. Nevertheless, studies are still scarce, findings are often inconsistent and with no difference according to the main substance of use [70]. Further studies are needed before evidence-based guidelines can be delivered for clinician's use.

### Myth 4: lithium treatment requires weekly lab tests

#### Fact 4: the optimal management of lithium treatment includes periodical laboratory tests

Lithium treatment can be easily implemented in ordinary clinical practice, both at inpatient and outpatient settings. Before starting treatment with lithium, it is recommended to assess blood concentrations of creatinine and urea-nitrogen (to check renal functioning), the levels of electrolytes (sodium, potassium, calcium), and the levels of thyroid and parathyroid hormones, as well as obtaining an electrocardiogram. Lithium blood concentration should be checked 5 days after the targeted dose is achieved. The evaluation of lithium blood concentration levels can be repeated until lithium reaches its therapeutic levels. Afterwards, lithium, creatinine, and TSH levels should be checked every one to 2 months in the first 6 months, and then every 6–12 months, or as clinically indicated.

The most common early side effects of lithium include reduced urinary concentrating ability (by 15% of normal maximum) with possible polyuria/polydipsia, tremor, and gastrointestinal symptoms. Clinical hypothyroidism and/or increased thyroid stimulating hormone (TSH), parathyroid abnormalities (usually normocalcemic hyperparathyroidism), impaired glomerular filtration rate and chronic kidney disease usually occur after a long-term exposure to lithium [75–77].

Some of lithium acute side effects can be reduced simply by ensuring that the lowest needed plasma levels are maintained and using prolonged-release formulations that are associated with lower plasma peaks.

In the long term, lithium is associated with a higher risk of impaired glomerular rate filtration and chronic kidney disease stage 3 (GFR < 60 mL/min/1.73 m^2^) as compared to valproate, olanzapine and quetiapine (but not CKD stage 4—GFR < 30 mL/min/1.73 m^2^); but lithium is significantly less associated with weight gain and hypertension than other mood stabilizers (e.g., valproate, olanzapine and quetiapine) [78]. Long-term antipsychotic exposure, especially some of the most used atypical antipsychotics such as olanzapine or quetiapine, is indeed associated with increased rates of obesity, metabolic syndrome and cardiovascular diseases, with an excess of mortality due to cardiovascular events [79]. Moreover, long-term exposure to dopamine-blocking agents may have an impact on brain’s reward system and on the extrapyramidal system.

In a small group of patients, lithium is associated with hypercalcemia or normocalcemic hyperparathyroidism (risk of reduced mineral density, osteoporosis and increased fracture risk over the long-term), but their clinical significance remains doubtful. In fact, opposite findings have been found recently, with decreased risk of osteoporosis (HRR, 0.62; 95% CI 0.53–0.72) in bipolar patients treated with lithium compared to those not receiving lithium. Treatment with antipsychotics, valproate, and lamotrigine was not associated with reduced risk of osteoporosis [80]. Concerning clinical hypothyroidism, annual TSH measurements may be sufficient to prevent overt hypothyroidism; in any case, thyroid function abnormalities should not constitute an outright contraindication to lithium treatment, as well as lithium should not be stopped if a responsive patient develops thyroid abnormalities [81, 82].

No studies have been performed to date evaluating the long-term effect of exposure to prolonged-release lithium versus immediate-release lithium on thyroid and renal function; however, one can expect that the lower peak plasma levels and the smaller peak-to-through plasma differences of the prolonged-release formulations are associated with a preserved thyroid and renal function.

Concerning adverse effects associated with lithium treatment, some clarifications need to be made. First, severe adverse renal and endocrine outcomes are very rare (although the absolute risk is higher and significant, the absolute number is low). According to a study in two regions of Sweden with 2.7 million inhabitants [83], renal replacement therapy in patients exposed to lithium occurs in 0.53% as compared to 0.08% in the general population (1.2% in those on lithium for more than 15 years), while a more recent study found that chronic kidney disease occurs in 0.6% of patients (median treatment time 19 months) [84]. Moreover, adverse renal consequences are associated with higher mean plasma levels (greater than 0.60–0.70 mEq/L) [85, 86], indicating that a regular monitoring of plasma levels can minimize or even neutralize the risk. Practical guidelines for prevention and management of renal side effects of lithium therapy advise to use a once-daily dosing schedule, which allow an effective treatment while preventing lithium intoxication [87].

### Myth 5: different formulations of lithium do not modify its tolerability profile

#### Fact 5: slow-release lithium formulation has advantages compared to immediate release formulation

Conventional slow-release (SR) lithium preparations provide a modulated release of the active ingredient, that is obtained for the reduced solubility of the saline compound used (lithium sulphate), or with the inclusion of lithium in a less easily absorbable matrix. In both cases, lithium is absorbed more slowly, and the peak plasma concentration, which is lower than the immediate-release (IR) formulations, occurs within 4–12 h; the pharmacokinetics, therefore, follow a plateau.

The slower increase in serum lithium concentrations and the lower Cmax (maximum concentration of drug detected in the blood) with SR formulations compared to IR lithium formulations translate into a reduced rate or less severity for some lithium-related adverse events, including tremors, upper gastrointestinal cramps, nausea, rash, cognitive obtundation, polyuria; a close relationship between changes in blood lithium levels and frequency and severity of side effects has been recently highlighted. Pompili et al. [88] found that SR lithium salts offer clinical advantages over IR formulations in terms of more stable circulating concentrations of lithium, less adverse impact on renal function, low incidence of adverse neurological effects (including cognitive impairment and tremor), low subjectively unpleasant adverse effects such as fatigue and weight-gain, and greater treatment adherence.

Slow-release formulations reduce the post-absorption peaks of plasma levels, which is beneficial for patients with gastric upset or transient side effects (e.g., tremors) secondary to temporary increases in blood levels.

Long-term treatment with SR lithium is associated with less impairment of the kidney's function to concentrate urine compared with IR lithium. A drug delivery system designed to achieve prolonged therapeutic effects by continuously releasing the medication over an extended time after the administration of a single dose reduces daily peaks in plasma lithium concentrations, thus preserving the functionality of the kidney.

A worsening of lower gastrointestinal disorders (e.g., diarrhea) has been occasionally reported with SR formulations of lithium compared to IR formulations [89].

A single daily administration of lithium can produce benefits compared to repeated daily administrations regarding adherence to therapy and reduction of renal and thyroid side effects. Double administration of lithium during the same day could produce renal impairment and a higher risk of polyuria [90]. A single evening dosage is recommended, allowing greater treatment adherence [38].

The overall decrease in adverse events with SR formulations compared to lithium IR formulations may reduce drug discontinuation, although this factor has not been evaluated in controlled clinical studies [90–93].

Prolonged-release lithium sulphate represents a therapeutic option of great advantage for the clinician, as it allows for constant plasma concentrations of lithium while minimizing the undesirable effects associated with concentration peaks of standard-release formulations [94]. Furthermore, in the event of an overdose of slow-release preparations, the chances of survival increase [95].

According to Bowden [89], adverse effects of lithium can be resolved by dose reduction, extended-release lithium, or combination therapy.

Barbuti et al. [96] investigated differences between patients receiving IR (lithium carbonate) vs. SR (lithium sulphate) lithium formulations in a prospective naturalistic study. Both lithium formulations significantly improved severity (CGI-BP scale) and functioning (FAST scale). These authors found a similar effect of lithium sulfate and lithium carbonate, but a lower incidence of tremors and gastrointestinal disturbances, as well as higher levels of adherence with lithium sulfate compared to lithium carbonate. Both formulations significantly improved both BD episode severity and functioning comparably to each other.

In a randomized clinical trial investigating the switch from IR to SR lithium formulation in poorly tolerant IR lithium-treated patients, Pelacchi et al. [97] found a significantly higher percentage of patients randomized to lithium sulfate (SR) vs. patients randomized to lithium carbonate (IR) who showed an improvement in tremor after the 1st week of treatment; and a persistence of tremor improvement after 12 weeks of treatment. Furthermore, a higher score for the «convenience» item (e.g., route of administration, dosing frequency) of the Treatment Satisfaction Questionnaire for Medication (TSQM scale) was reported by patients randomized to lithium sulfate vs. those randomized to lithium carbonate.

These benefits have led to a frequent use of the extended-release formulation of lithium in many European countries. Still, maybe due to the minimal publicity of these benefits, relatively less use has occurred in other parts of the world.

### Myth 6: no drug has an antisuicidal effect

#### Fact 6: lithium treatment has antisuicidal properties

Lithium is recommended for the reduction of suicide risk among patients with bipolar disorder [98]. The first evidence of lithium’s antisuicidal properties date back to the seminal studies by Barraclough [99], pointing out that lithium was effective in preventing “recurrent affective illness”. Jamison [100] postulated that lithium would demonstrate its role in preventing suicide in bipolar disorders in the next 10 years. Several studies reported strong evidence of the role of lithium in suicide risk protection among patients suffering from mood disorders. The fatality rate was 8.7 times lower during versus after discontinuing lithium and gradual vs. rapid discontinuation of lithium could limit the risk of suicidal behavior [101].

A significant reduction in suicidal risks (attempts > suicides) with lithium maintenance therapy in unipolar ≥ bipolar II ≥ bipolar I disorder to overall levels close to general population rates was found in people treated with lithium. Patients who did not receive lithium had alarming rates of both suicides and suicide attempts in all types of the above-mentioned mood disorders [102].

Patients suffering from bipolar disorder or any other major affective disorder treated with lithium reported a risk of completed and attempted suicides lower than 80% compared with patients treated with other drugs (risk ratio = 4.91, CI 3.82–6.31; *P* < 0.0001) [103].

A potentially important new observation is a strong association of lithium treatment with the ratio of attempted to completed suicides, which was proposed as an index of the lethality of suicidal acts. In the available literature, the attempted suicide/completed suicide ratio (A/S) is 2.5 times greater among lithium-treated subjects than in those not treated with lithium, and nearly three times higher among bipolar disorder cases, suggesting a reduction in lethality attributable to lithium treatment with fewer fatalities per attempt.

Lithium can have an independent antisuicidal effect because of suicidal behavior in a selected group of high-risk mood disorder patients. Evidence supports the notion of a significantly decreased rate of suicide attempts compared to pre-lithium figures, not only in patients with excellent treatment outcomes, but also in those with moderate or poor response to lithium prophylaxis [104]. These findings highlight a specific antisuicidal action of lithium on the suicide risk dimension, independent from the clinical mood disorder picture [105]. Cipriani et al. [106] found that lithium effectively prevents suicide, deliberate self-harm, and death from all causes in patients with mood disorders. The anti-suicidal properties of lithium in mood disorders have been more recently confirmed [107, 108].

However, some studies did not find consistent antisuicidal action of lithium salts. For example, a randomized controlled trial did not find differences between lithium and valproate in preventing suicide attempts and suicide events in bipolar disorder patients [109]. Another randomized placebo-controlled clinical trial found that adding lithium to treatment does not reduce the incidence of suicide-related events in veterans with major depressive disorder or bipolar disorder who experienced a recent suicide event. In addition, they observed that adding lithium to existing medication regimens does not prevent a broad range of suicide-related events in patients who were being treated for mood disorders and previously engaged in suicidal behavior [110]. However, such exceptions seem irrelevant when considering that nearly three dozen observational trials have found fewer suicides or attempts in patients treated with lithium than without; that there are similar effects in several randomized clinical trials in which suicidal behaviors were considered adverse events rather than an explicit outcome, and that there is marked, temporary increases in suicidal behavior soon after discontinuing lithium treatment [111].

Furthermore, the use of lithium as a lethal mean for suicide has not received support, as data indicate that mortality from an overdose of lithium is similar to that associated with other psychotropic drugs, or even less, as well as suicidal gestures with lithium are uncommon, or even absent, during long-term lithium treatment [98]. The availability of slow-release formulation may reduce side effects and improve adherence [88, 97].

Lithium has the potential to act trans-diagnostically, given evidence emerging from studies of lithium in drinking water and the rates of suicide risk in selective areas with higher concentrations [112]. Despite the controversy, drinking lithium-rich water may protect general populations from suicide. As hospitalizations are a critical time for mood disorders patients, especially at discharge for the increased suicide risk, it is noteworthy to mention a recent study [113] that demonstrates a marked overall reduction in hospitalization during lithium treatment compared to the year before starting lithium, both for bipolar disorder and major depressive disorder.

### Myth 7: lithium treatment should be avoided during pregnancy

#### Fact 7: lithium can be carefully managed during pregnancy

The decision to treat mood disorders during the peripartum period should be carefully considered, balancing the risk of prenatal and neonatal exposure to the medication versus the potentially deleterious effects of untreated affective episodes on the maternal and neonatal health. Accumulating evidence shows that the continuation of prophylactic medication with mood stabilizers (particularly lithium) during the perinatal period may prevent affective recurrences.

Lithium should be considered the first-choice treatment for bipolar disorder in the peripartum period, especially in women already treated and stabilized with lithium before pregnancy.

Stevens and colleagues [114] demonstrated that the rates of peripartum mood relapses in bipolar disorder are significantly higher if stabilizing therapy is discontinued before or during pregnancy (68%) compared to its continuation (23%). The recurrence risk is much increased if the cessation of the mood stabilizer is rapid and abrupt (within 14 days): about half of women who suspended suddenly or quickly lithium experienced a mood recurrence within 2 weeks [115]. Instead, the maintenance of therapy with lithium reduces the relative risk of relapse by 66% [114].

The efficacy/safety ratio seems to be in favour of lithium in respect to other mood stabilizers in the prophylactic treatment of pregnant women with bipolar disorder. The use of lithium during pregnancy is very effective in preventing depressive and (hypo)manic episodes and associated with few side effects on fetal and newborn health [116]. During pregnancy, the administration of lithium may be correlated with an increased risk of cardiac malformations (2.1% among lithium-exposed pregnancies vs 1.6% among non-exposed), but the difference is not significant [117]. Especially, lithium exposure may induce the development of Ebstein’s anomaly, affecting 5–7 infants per 1.000 live births (instead of 1 per 20.000 live births in general population) [118]. The risk of developing heart defects is associated with the time of lithium intake (higher in the first-trimester of pregnancy) and dosage exposure (lower if serum lithium levels < 0.64 mEq/L and dosages < 600–900 mg/day) [118, 119]. Lithium exposure during the first trimester of pregnancy seems to be associated with higher rates of spontaneous abortion compared with those in general population; however, the risk of miscarriage appears similar when comparing with lithium-unexposed patients with affective disorders [119].

The administration of lithium treatment does not contraindicate breastfeeding because of the absence of severe adverse events reported in infants exposed through maternal milk [120]. Few studies show rare and transient lithium toxicity (e.g., hypotonia, weight loss in the first week, thyroid and renal parameter alterations) in breastfed infants, which recovers after discontinuation of lactation [121]. Lithium salts can be taken during breastfeeding, but a frequent evaluation of infant serum levels and a strict paediatric monitoring are mandatory [122]. However, the decision to breastfeed should also be evaluated in relation to the risk of affective relapses correlated to sleep dysregulation [123].

Some caution must be used in the stabilizing treatment of pregnant women. Preferentially, lithium should be prescribed as monotherapy, in fractionated doses or in slow-release formulations, to avoid plasma peak concentrations and within the lowest therapeutic range throughout pregnancy, particularly during the first trimester. Furthermore, lithium dosages can be reduced immediately before delivery to reduce the risk of postnatal adaptation syndrome [124]. Because of changes in the mother's plasma volume and renal clearance through pregnancy, a close monitoring of lithium blood levels is recommended: lithium blood levels decrease in the first trimester, remains stable in the second trimester, increase in the third trimester and are still slightly increased after delivery [125]. In addition, supplementation with folic acid (5 mg/day) in pregnancy reduces the risk of foetal and neonatal negative outcomes [122].

## Discussion

Personalized and integrated treatment, including pharmacological and non-pharmacological interventions, should be used in patients with bipolar disorder. Although several pharmacological treatments are indicated as first-line treatment for the management of patients with bipolar disorder, lithium is the gold standard due to its established mood-stabilizing properties [23, 24, 126].

Lithium is one of the most effective treatments available in psychiatry [25], and psychiatrists should be aware of the great applicability of lithium use, considering its efficacy and good tolerability profile. However, the recent years have witnessed a decreasing trend in the use of lithium in clinical practice. Many early career psychiatrists or recently graduated physicians do not feel skilled enough to use lithium, which is considered a difficult-to-use agent, mainly for its subtleties, the need for periodical blood checking, and its side effects [29]. Moreover, several stereotypes related to the difficulties in managing lithium treatment exist, which further reduce the use of lithium in clinical practice. Thus, in this narrative review we aim to overcome some of these misconceptions regarding lithium, by counterarguing myths with facts.

Lithium is an excellent treatment for patients suffering from bipolar disorder. In particular, lithium is a valid treatment option in the different phases of bipolar disorder, including treatment of current manic/hypomanic episodes, especially in combination with second-generation antipsychotics. A recent review of all clinical practice guidelines for the treatment of patients with bipolar disorder highlights that lithium is recognized as a key pharmacotherapeutic agent in the treatment of bipolar disorder [127].

Lithium has also been found to have an outstanding efficacy in the maintenance or prophylactic treatment of bipolar disorder [128, 129], representing the gold standard treatment for the long-term prevention of affective relapses in patients with bipolar disorder [130]. In both controlled clinical trials and in observational studies, lithium has shown its efficacy and superiority in the prophylaxis of any type of affective episode [39, 131–135].

One important misconception regarding lithium is that it should be avoided in adolescents or elderly patients due to its side effects. This notion has been counteracted by several clinical studies, which have highlighted that lithium is well-tolerated and effective both in adolescents and in elderly patients with bipolar disorder [46, 136]. In particular, the recent umbrella review by Janiri et al. [45] found that lithium is superior to placebo in pediatric and adolescent patients with bipolar disorder. All studies specified that lithium was generally well tolerated in this group of patients, with common side effects (e.g., gastrointestinal, polyuria or headache) similar to those reported in adults. As regards the use of lithium treatment in elderly patients with bipolar disorder, De Fazio et al. [136] found that lithium was superior to placebo and to other mood stabilizers in treating mania. The efficacy of lithium in geriatric patients with resistant major depressive disorder is supported also by Ross [137] and by Cooper et al. [138]. Regarding lithium tolerability in elderly patients, the clear evidence is that lithium may be relatively well-tolerated, but low doses should be used in the elderly, since the risk of adverse events increases according to a dose-dependent pattern.

Many early career psychiatrists frequently report to not prescribe lithium treatment since they feel not enough skilled in detecting and managing the potential drug–drug interactions and their impact on patients. This misconception highlights the need for an adequate training of prescribers on the potential pharmacodynamic and pharmacokinetic interactions of lithium. Improving knowledge and confidence with lithium monitoring can significantly contribute to improve patient outcomes and increase lithium use in ordinary clinical practice. The risk of potential drug–drug interactions can be easily monitored in patients treated with lithium, also considering that the risk increases when patient gets older because of declining renal function and accumulation of medical comorbidities [139]. Other conditions requiring a careful evaluation of lithium treatment are those causing haemodynamic and volume alterations such as dehydration, fever, gastrointestinal loss, perioperative management and surgery. Prescribers should be aware that the most common (potential) interactions occur with ACE inhibitors, angiotensin II receptor antagonists (sartans), diuretics, and NSAIDs. Combinations of these drugs are frequent, so clinicians—being aware of their additive effects—should regularly check lithium concentration, not excluding lithium treatment. As suggested by Malhi et al. [140], “lithiumeter”, which is a visual and practical guide for determining lithium levels in the management of bipolar disorder, is a useful tool for supporting the use of lithium treatment in ordinary clinical practice. Furthermore, providing patients with correct information on the benefits of lithium treatment is essential to improve their adherence to the prescribed therapy [141–143].

Another common stereotype is that using different lithium formulations does not modify lithium tolerability. On the contrary, the availability of prolonged-release lithium formulation represents an important strength of lithium treatment choice since it reduces some of the most common side effects of lithium, such as tremor and gastrointestinal symptoms, and also has a positive effect on adherence [96, 97].

A relevant statement clearly emerging by our review is that lithium has antisuicidal properties, which might be unrelated to its mood-stabilizing effects, considering that suicidality is a complex phenomenon, with multifactorial causes [144–148]. In fact, there is a significant reduction in the number of suicide attempts among individuals who take lithium, even among those who do not respond well to the prophylaxis of mood symptoms with lithium. The anti-suicidal properties of lithium appear to be as effective at low concentrations as at therapeutic levels. Sustained low doses of lithium intake have shown to decrease suicidality. Suicide prevention in patients taking lithium occurs when the serum concentrations are within a therapeutic range of 0.5–1.0 mmol/L. The lowest efficacious lithium level in the long-term treatment of patients with bipolar disorder has been shown to be 0.4 mmol/L, with optimal response achieved at concentrations between 0.6 and 0.75 mmol/L. Those with manic symptoms might benefit from higher levels of lithium concentration. The duration of lithium treatment is also important for suicide prevention. Even among patients with only poor-to-moderate clinical response, a decrease in the number of suicide attempts has been observed when patients take lithium compared to those who do not take it. Research supports the recommendation that lithium treatment should be considered for anyone at high risk for suicide, even if mood-stabilization is not achieved [149, 150].

Finally, a very controversial issue is the use of lithium in pregnancy. The review by Poels et al. [151] reported lower risk estimates of the association between first trimester lithium exposure and risk of congenital malformations than previously reported. Moreover, no association between lithium use and pregnancy or delivery-related outcomes were found, although studies are needed. Therefore, lithium is effective and relatively safe in pregnancy and postpartum for the prevention of relapse in bipolar disorder; during the first trimester, tapering of lithium treatment should be considered weighing benefits against relapse risks. Accumulating evidence shows that the continuation of prophylactic medication with mood stabilizers (particularly lithium) during the perinatal period may prevent affective recurrences. In particular, Stevens et al. [114] demonstrated that the rates of peripartum mood relapses in bipolar disorder are significantly higher if stabilizing therapy is discontinued before or during pregnancy. When lithium is prescribed during pregnancy, some caution must be used, and lithium blood levels should be monitored more frequently, preferably weekly in the third trimester.

Finally, given its neuroprotective and neurotrophic properties, lithium improves cognition, either associated or not to an acute affective episode [152, 153]. Increasing evidence indicates lithium efficacy in neuroprotection, since it restores both neurotransmission and brain structure [154]. The recognition of lithium as neuroprotective agent is explained by the discoveries of several key cell signaling-related enzymes activated by lithium treatment. These include glycogen synthase kinase-3, inositol monophosphatase, phosphoadenosylphosphate phosphatase, and Akt/beta-arrestin 2. Processes associated with these enzymes include autophagy, BDNF cellular signaling, neuroinflammation, mitochondrial function, and apoptosis. The involvement of these processes in a variety of brain disorders beyond bipolar disorders, such as stroke, Huntington’s disease, Alzheimer’s disease, Parkinson’s disease, fragile X syndrome, amyotrophic lateral sclerosis, and multiple sclerosis raised the interest in exploring lithium’s potential neuroprotective property in these neurodegenerative and neurodevelopmental disorders [155].

The present study has some limitations which must be acknowledged. Firstly, the consensus paper is based on a narrative review of the available literature on efficacy, tolerability and use of lithium in ordinary clinical practice. The search has not adopted a rigorous methodology, analyzing all available databases and sources of information. Moreover, a specific focus on myths/facts related to lithium treatment with a specific focus on adolescent/young patients is missing, since other recent papers have already assessed the use of lithium in such target group.

## Conclusions

In recent years, a discrepancy between evidence-based recommendations and clinical practice in using lithium treatment for patients with bipolar disorder has been highlighted.

It is time to disseminate clear and unbiased information on the clinical efficacy, effectiveness, tolerability and easiness to use of lithium treatment in patients with bipolar disorder. The availability of different formulations of lithium represents a further strength of this therapeutic choice, since the treatment can be more easily adapted to patients’ needs.

It is necessary to reinvigorate the clinical and academic discussion about the efficacy of lithium, to counteract the decreasing prescription trend one of the most effective drugs available in the whole medicine.

## Data Availability

Upon request to corresponding author.
